# Genomic Analyses Provide Insights Into the Evolutionary History and Genetic Diversity of *Auricularia* Species

**DOI:** 10.3389/fmicb.2019.02255

**Published:** 2019-10-01

**Authors:** Yueting Dai, Xiao Li, Bing Song, Lei Sun, Chentao Yang, Xin Zhang, Yanfeng Wang, Zhiwu Zhang, Yongping Fu, Yu Li

**Affiliations:** ^1^Engineering Research Center of Chinese Ministry of Education for Edible and Medicinal Fungi, Jilin Agricultural University, Changchun, China; ^2^China National GeneBank, BGI-Shenzhen, Shenzhen, China; ^3^Mudanjiang Branch of Heilongjiang Academy of Agricultural Sciences, Mudanjiang, China; ^4^Department of Crop and Soil Sciences, Washington State University, Pullman, WA, United States; ^5^Internationally Cooperative Research Center of China for New Germplasm Breeding of Edible Mushroom, Jilin Agricultural University, Changchun, China

**Keywords:** Wood Ear, *Auricularia cornea*, genome sequencing, genetic variation, molecular marker

## Abstract

Species in the genus *Auricularia* play important roles for people’s food and nutrition especially *Auricularia cornea* and *A. heimuer*. To understand their evolutionary history, genome structure, and population-level genetic variation, we performed a high-quality genome sequencing of *Auricularia cornea* and the corresponding comparative genomic analysis. The genome size of *A. cornea* was similar to *Auricularia subglabra*, but 1.5 times larger than that of *A. heimuer*. Several factors were responsible for genome size variation including gene numbers, repetitive elements, and gene lengths. Phylogenomic analysis revealed that the estimated divergence time between *A. heimuer* and other *Auricularia* is ∼79.1 million years ago (Mya), while the divergence between *A. cornea* and *A. subglabra* occurred in ∼54.8 Mya. Population genomic analysis also provided insight into the demographic history of *A. cornea* and *A. heimuer*, indicating that their populations fluctuated over time with global climate change during Marine Isotope Stage 5-2. Moreover, despite the highly similar external morphologies of *A. cornea* and *A. heimuer*, their genomic properties were remarkably different. The *A. cornea* genome only shared 14% homologous syntenic blocks with *A. heimuer* and possessed more genes encoding carbohydrate-active enzymes and secondary metabolite biosynthesis proteins. The cross-taxa transferability rates of simple sequence repeat (SSR) and insertion or deletion (InDel) markers within the genus *Auricularia* were also lower than that previously observed for species within the same genus. Taken together, these results indicate a high level of genetic differentiation between these two *Auricularia* species. Consequently, our study provides new insights into the genomic evolution and genetic differentiation of *Auricularia* species that will facilitate future genetic breeding.

## Introduction

*Auricularia* fungi naturally grow on wood, logs, branch and twigs of trees and generally distribute in China, Africa, America, and Australia, etc (Wu et al., 2014, 2015). Among this genus, *A. cornea* and *A. heimuer* have been domesticated and cultivated for more than 1,400 years, and given the commercial name Wood Ear (Zhang et al., 2015). Currently, Wood Ear is widely commercialized, particularly in China, with an annual production over the last 5 years of approximately eight million tons. A variety of agro-industrial wastes can be used to cultivate Wood Ear, such as sawdusts, crop straw, and cottonseed hulls (Sánchez, 2010; Abd Razak et al., 2012; Zhang et al., 2016). As one of the edible and medicinal mushrooms, Wood Ear exhibits beneficial health effects such as anti-hypercholesterolemic (Zhao et al., 2015), antioxidant, and antimicrobial properties (Cai et al., 2015; Avci et al., 2016). Therefore, it is important to develop the genetic-based resources of Wood Ear to better understand its evolutionary history, domestication and medicinal benefits, as well as improve its utilization.

Among Wood Ear species, the genomes of *A. heimuer* and *A. subglabra* have been sequenced (Floudas et al., 2012; Yuan et al., 2019). Based on the genomic and comparative transcriptomic analysis of *A. heimuer*, Yuan et al. (2019) identified some genes that were putatively associated with wood degradation, the biosynthesis of bioactive proteins and polysaccharides. In addition, some genes putatively related to fruiting body development were identified with *de novo* transcriptome assembly of *A. cornea*, along with additional detection of expressed sequence tag-simple sequence repeat (EST-SSR) loci (Zhou et al., 2014). Some other kinds of molecular markers also have been developed for Wood Ear, such as random amplified polymorphic DNA (RAPD) (Yan et al., 2004), sequence-related amplified polymorphisms (SRAPs) (Yu et al., 2008; Du et al., 2011), inter-simple sequence repeats (ISSR) (Yu et al., 2008; Du et al., 2013), and SSR (Zhang et al., 2012). The two single-nucleotide polymorphism (SNPs) have been identified that could be associated with regulation of the fruiting body colors of *A. cornea* (Huang et al., 2018). However, the genome-wide variation maps for Wood Ear are not constructed using the whole genome re-sequencing technology. Moreover, the lack of a high-quality reference genome for *A. cornea* limits its genetic research and our understanding of the evolutionary history of *Auricularia*.

In this study, *de novo* genome sequencing of *A. cornea* was conducted on the single-molecule real-time (SMRT) sequencing platform followed by whole genome resequencing of 24 strains using the Illumina Hiseq X Ten sequencing platform. The specific objectives of this study were to: (1) explore the genomic evolution and genome features of *A. cornea* and *A. heimuer*; (2) identify the genomic polymorphisms in *A. cornea* and *A. heimuer* populations and estimate their demographic history; and (3) develop novel SSR and InDel markers for *A. cornea*.

## Materials and Methods

### *Auricularia* Strains

*De novo* genome sequencing was conducted on the monokaryon *A. cornea* strain AC1 (hereafter, AC1) that was derived from the cultivated strain CCMJ2567 using protoplast monokaryogenesis technique (Dai et al., 2017), but with a lywallzyme incubation time of 5 h. A total of 24 Wood Ear strains were used for resequenced the whole genome, including 12 *A. cornea* strains (seven cultivated strains from China and five wild individuals from Africa) and 12 *A. heimuer* strains from China (six cultivated strains and six wild individuals) (Supplementary Table S1). These strains were cultured on Malt Yeast Extract Glucose (MYG) medium (10 g/L maltose, 5 g/L yeast extract powder, and 5 g/L glucose) with cellophane sheets and then incubated at 24°C for 9 days. The mycelia were then harvested and quickly frozen in liquid nitrogen. Genomic DNA of each strain was extracted using the NuClean Plant Genomic DNA Kit (CWBIO, Beijing, China) according to the manufacturer’s protocols. DNA quality, purity, and concentration were then evaluated using agarose gel electrophoresis, NanoDrop spectrophotometry (Thermo Fisher Scientific, Waltham, MA, United States), and Qubit fluorometry (Life Technologies, CA, United States), respectively. These Wood Ear strains were maintained at the Engineering Research Center of Chinese Ministry of Education for Edible and Medicinal Fungi (ERCCMEEMF) at the Jilin Agricultural University (Changchun, China).

### *De novo* Genome Sequencing, Assembly, and Annotation

*De novo* genome sequencing of AC1 was conducted on the Pacific Biosciences (PacBio) Sequel platform at ERCCMEEMF. The sequencing library was prepared by mechanically shearing 8 μg of gDNA with g-TUBE devices (Covaris, MA, United States). The 20 Kbp SMRTbell DNA library was generated using the BluePippin DNA size selection protocol (Sage Science, MA, United States). SMRT sequencing of longer reads was conducted with four SMRT cells following the manufacturer’s protocols. Subreads were assembled using the open source SMART *de novo* assembly pipeline^1^. Mapping of the conserved genes was conducted to estimate genome completeness using the Core Eukaryotic Genes Mapping Approach (CEGMA) (Parra et al., 2007) and Benchmarking Universal Single-Copy Orthologs (BUSCO) pipelines (Simão et al., 2015; Waterhouse et al., 2018). The final assembled *A. cornea* strain AC1 genome was deposited in GenBank (RJDY00000000). Repeat element, coding and non-coding gene prediction, along with functional annotation of the AC1 genome assembly, were all conducted as described previously (Wang et al., 2019). The assembly contigs were firstly masked using RepeatMasker^2^. The unmasked sequences were then used to search for repeat elements using RepeatModeler^3^. Meanwhile, the repeat-masked AC1 genome was used for gene predictions, which combined the *ab initio* and protein homology-based prediction methods. Among them, SNAP (Korf, 2004), Augustus (Stanke et al., 2006), Genescan (Burge and Karlin, 1997), and GlimmerHMM (Majoros et al., 2004) were used for *ab initio* gene predictions. For homologous protein mapping, protein sequences of *Schizophyllum commune* (Ohm et al., 2010), *Coprinopsis cinerea* (Stajich et al., 2010), *Agaricus bisporus* (Morin et al., 2012) and *Pleurotus ostreatus* (Qu et al., 2016) were downloded from the National Center for Biotechnology Information (NCBI) database and aligned using tBLASTn and Genewise (Birney et al., 2004). For non-coding genes, transfer RNA (tRNA), ribosomal RNA (rRNA), microRNA (miRNA) were predicted using tRNAscan-SE (Lowe and Eddy, 1997), RNAmmer (Lagesen et al., 2007) and Rfam (Gardner et al., 2009), respectively. Moreover, the MIcroSAtellite (MISA) tool was used to identify microsatellite loci^4^.

Gene function predictions were according to the best alignment attained using BLASTP to NCBI non-redundant database (Nr), Eukaryotic Clusters of Orthologous Groups (KOG) (Tatusov et al., 2004), and UniProt database (including SwissProt and TrEMBL databases) (Bairoch and Apweiler, 2000) with *e*-value <1e-5. The gene models were also annotated by their protein domains using InterPro database (Mitchell et al., 2015). On the basis of Nr and InterPro databases, Blast2go was used to classify all genes by Gene Ontology (GO) (Ashburner et al., 2000). Additionally, Kyoto Encyclopedia of Genes and Genomes (KEGG^5^) pathway annotation of genes was performed using the BLASTX algorithm with *E*-values <1e-5 (Kanehisa et al., 2004). Furthermore, the published genome sequence of *A. heimuer* ASM228711 (Yuan et al., 2019) was re-annotated (hereafter, ASM) using the same methods as described above. Then, all the predicted protein sequences in AC1 and ASM genomes were subjected to the carbohydrate-active enzyme (CAZyme) database to search against sequence libraries with the families of glycoside hydrolases (GHs), auxiliary activities (AAs), carbohydrate-binding modules (CBMs), glycosyltransferases (GTs), polysaccharide lyases (PLs), and carbohydrate esterases (CEs). The gene clusters associated with secondary metabolic biosynthesis was searched using Antibiotics & Secondary Metabolite Analysis Shell (antiSMASH^6^).

### Evolutionary Analysis of *Auricularia*

The construction of a phylogenetic tree for *A. cornea*, *A. heimuer*, and other 19 published fungal species were conducted as previously described (Wang et al., 2019) including *A. subglabra* (AS) TFB-10046 SS5, *Tremella mesenterica* DSM 1558, *Coniophora puteana* RWD-64-598 SS2, *Trametes versicolor* FP-101664 SS1, *Dacryopinax primogenitus* DJM 731 (Floudas et al., 2012), *Laccaria bicolor* S238N-H28 (Martin et al., 2008), *Serpula lacrymans* S7.9 (Eastwood et al., 2011), *C. cinerea* okayama7#130 (Stajich et al., 2010), *S. commune* H4-8 (Ohm et al., 2010), *Ganoderma sinense* ZZ0214-1 (Zhu et al., 2015), *Tremella fuciformis*^7^, *Exidia glandulosa* HHB12029, *Sistotremastrum niveocremeum* HHB9708 (Nagy et al., 2015), *Phellinus lamaoensis*, *Coniferiporia sulphurascens* (Chung et al., 2017), *Polyporus brumalis* (Miyauchi et al., 2018), *Lentinus tigrinus* ALCF2SS1-7 (Wu et al., 2018), *Trametes hirsute* (Pavlov et al., 2015), *Sparassis crispa* (Kiyama et al., 2018). The common, unique, and single-copy gene families of these 21 species were identified with OrthoMCL (Li et al., 2003) and OrthoVenn (Wang et al., 2015). Then, the single-copy gene families were used to construct the phylogenetic tree for these species. The fossil calibration points were selected using previously described methods (Floudas et al., 2012). The expansion and contraction of gene families were identified using Computational Analysis of gene Family Evolution (CAFE) (De Bie et al., 2006), which were set to *p*-value cutoff of 0.05, number of random samples = 1000, and search for the λ value. The positive selection was detected using PAML^8^. Then, the selected genes were functional annotated with GO and KEGG databases. Furthermore, whole-genome collinearity analysis of *A. cornea* and *A. heimuer* was conducted using the MCscan program^9^.

### Resequenced Whole Genomes of *A. cornea* and *A. heimuer* Strains

The whole genome resequencing of 24 Wood Ear strains were conducted on the Illumina HiSeq X Ten platform at Novogene Co., Ltd. (Beijing, China). For each strain, 1.0 μg of genomic DNA was used to generate 350 bp sequencing library. Low-quality reads with adapter sequences and duplicate reads were firstly filtered using SAMtools^10^. The obtained high-quality clean reads were then mapped to the AC1 and ASM genomes using the Burrows-Wheeler Aligner (BWA) tool (Version: 0.7.8) (Li and Durbin, 2009). The mapped reads were used to identify SNP and InDel polymorphisms with the Genome Analysis Toolkit (GATK) (Version: 3.8.0) program using default parameters (McKenna et al., 2010). All variants were annotated using the ANNOVAR program (Wang et al., 2010). The effective population sizes of AC1 and ASM were also estimated using the Pairwise Sequentially Markovian Coalescent (PSMC) method (Li and Durbin, 2011). In these analyses, the generation time (g) was set as 1 year, and the mutation rate (μ) was set to 3.4 × 10^–9^. Furthermore, the genome-wide selection test of the Asia and Africa populations of *A. cornea* was conducted. The fixation index (F_*ST*_) of these two population was estimated by PoPoolation2 software (Kofler et al., 2011) and Sliding Window Algorithm (step size 500 bp, window size 5,000 bp). Selected regions were measured based on Z transformation of F_*ST*_ (ZF_*ST*_, threshold values more than four). The selection regions were then functional annotated using GO and KEGG database.

### Design and Validation of Novel SSR and InDel Markers for *A. cornea*

SSR and InDel primer pairs for *A. cornea* were designed using Primer3^11^. A total of 50 SSR and 50 InDel primer pairs were selected to determine polymorphism levels within *A. cornea* strains. The primers were synthesized by Beijing Dingguochangsheng Biotech Co., Ltd. (Beijing, China). PCRs were then conducted in reaction conditions and thermal cycling protocols as described previously (Fu et al., 2017), but with an annealing temperature of 59°C. PCR products were confirmed using silver staining (Dai et al., 2017).

## Results

### Sequencing and Assembly of the *A. cornea* Genome

**FIGURE 1:**
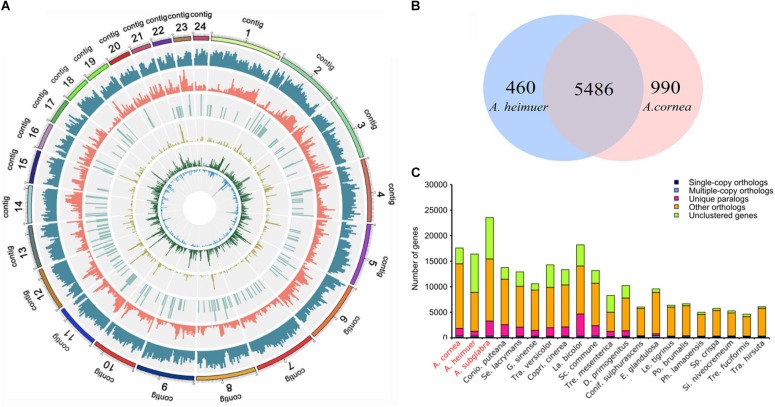
Genome map and gene family analysis of the *A. cornea* genome. **(A)** Genome map of *A. corne*a. Outside to inside of concentric circles show assembly contig number, gene density, all repeat content, non-coding RNA (ncRNA), LINE content, LTR content, and DNA repeat content of AC1 genome. **(B)** Unique and share gene families in the genomes of *A. cornea* AC1 and *A. heimuer* ASM. The number of unique and shared gene families is shown in each of the diagram components. **(C)** Comparison of orthologous genes among the genomes of 21 fungal species.

To obtain a high-quality reference genome for *A. cornea*, we performed *de novo* genome sequencing of the monokaryon strain AC1 using PacBio Sequel platform (Table 1). A total of 22.37 Gbp (304 × genome coverage) of single-molecule long reads were generated and *de novo* assembled. The assembled AC1 genome comprised 24 contigs (contig N50 = 4.35 Mbp) with an estimated genome size of 73.48 Mbp and a GC content of 59.58% (Figure 1A). Moreover, 91.13% coverage of core eukaryotic genes and 96.20% coverage of single-copy orthologs were exhibited in the AC1 genome, as estimated by the CEGMA and BUSCO programs, respectively. Taken together, these results indicate the generation of a high-quality *A. cornea* AC1 genome assembly.

**TABLE 1:** Summary of assembly and annotation statistics for the genomes of *A. cornea* and *A. heimuer*.

**Assembly feature**	***A. cornea* (AC1)**	***A. heimuer* (ASM)**
Sequencing method	PacBio Sequel	PacBio RSII
Genome size (Mbp)	73.48	49.76
Coverage (X)	304	56.97
Number of contigs	24	103
N50 (Mbp) of contigs	4.35	1.35
GC content (%)	60	57
Repeat abundance (%)	21.73	18.58
LTR abundance (%)	10.33	8.99
Gene number	17,591	16,402
Average gene length (bp)	2,541	2,176
Average exon length (bp)	308	284
Average intron length (bp)	217	291
Average number of exons per genes	5.26	4.29

### Gene Prediction and Annotation for the AC1 and ASM Genomes

To avoid systematic biases from different methodologies, we annotated the AC1 and ASM genomes using the same methods based on the uniform reference gene sets. According to the combination of homology-based and *de novo* prediction methods, a total of 15.96 Mbp (21.73%) and 9.24 Mbp (18.58%) sequence regions in the AC1 and ASM genomes were comprised repeat elements, respectively. Most types of transposable elements (TEs) were identified in these two genomes, including long terminal repeat (LTRs) retrotransposons that were most abundant type, LINE retrotransposons, and DNA transposons. The Non-coding RNAs in the two genomes were with similar copy numbers. For example, the AC1 genome contained 14 micro RNA (miRNAs), 143 transfer RNAs (tRNAs), 25 ribosomal RNAs (rRNAs) and 21 small nuclear RNAs (snRNAs). Likewise, the ASM genome contained 38 miRNAs, 138 tRNAs, 33 rRNAs and 19 snRNAs.

According to the *de novo* and homology-based prediction methods, a total of 17,591 and 16,402 protein-coding genes were predicted for the AC1 and ASM genomes, respectively (Table 1). The average length of the coding sequences in the AC1 genome was 1,618 bp, which contained five exons with an average length of 308 bp, while those of in the ASM genome exhibited an average length of 1,218 bp containing an average of four exons with an average length of 284 bp. A total of 16,569 (94%) and 10,930 (67%) genes in the AC1 and ASM genomes were homologous to known proteins in the eight databases (Supplementary Table S2).

We then identified a total of 4,168 and 2,663 SSR loci in the AC1 and ASM genomes, respectively. The density of SSR loci was similar among these two genomes, after accounting for differing genome sizes. Trinucleotide repeats (TNR) were the most common type accounted for 40% of the total loci in the two genomes (Supplementary Table S3), followed by dinucleotide repeats (DNR, 29–40%). The other types were only individually accounted for 2–4% of the total genome sizes. Tandem repeats with five repeats were the most common TNR repeat types in the two genomes, followed by tandem repeats with six repeats of DNR and TNR. Among SSR types, CG, CCG, TACC, GGTTA, ACGGCG, and CTCTTC were the most prevalent forms for corresponding repeat types in the AC1 genome, while GC, CAG, GGTA, CCTAA and CCGCAG were most prevalent in the ASM genome.

### Comparative Genomic and Evolutionary Analysis for *A. cornea* and *A. heimuer*

**FIGURE 2:**
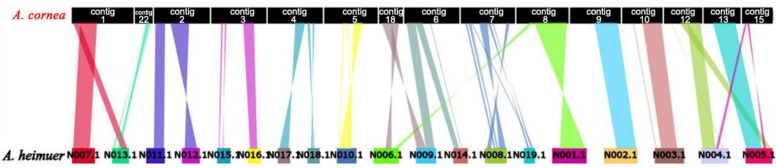
Whole-genome collinearity analysis of *A. cornea* and *A. heimuer*.

The assembled size of AC1 genome was 73.48 Mbp larger than ASM (49.76 Mbp). A total of 4,163 single copy genes (Figure 1C) and 5,486 homologous gene families were shared by AC1 and ASM, while 990 and 460 unique gene families were specific to each genome, respectively (Figure 1B). Analysis of collinear genomic regions between AC1 and ASM revealed very few conserved syntenic blocks (10.60 Mbp) between the two genomes, which was accounting for 14 and 21% in AC1 and ASM, respectively (Figure 2).

**FIGURE 3:**
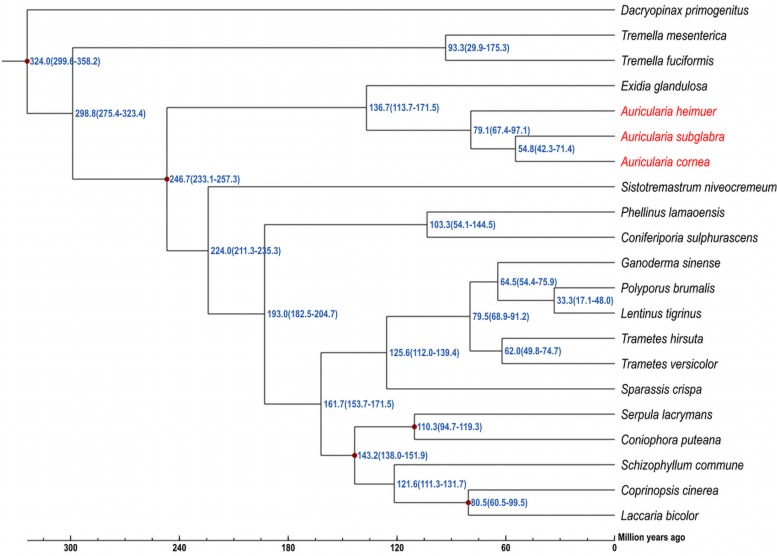
Phylogenomic analysis of 21 fungal genomes. The estimated divergence times are shown at the nodes, and the overall timeline is shown below the phylogenetic tree.

To further understand the genomic evolution of *A. cornea* and *A. heimuer*, we selected 19 reported fungal genomes presenting three class, eight orders, 12 families to conduct the phylogenetic analysis. A total of 56 single-copy orthologous genes were shared among these 21 genomes, which were consequently used to construct a phylogenetic tree with the maximum likelihood (ML) method. The phylogenetic analysis indicated that four species in Auriculariales were clustered in one branch. Among them, three species in genus *Auricularia* were clustered as a subgroup, which *A. cornea* was most closely related to *A. subglabra* and then clustered with *A. heimuer*. The divergence time of Tremellomycetes and Agaricomycetes was estimated as ∼298.8 (275.4–323.4) Mya. Auriculariales was estimated to have a divergence time with other five orders (Trechisporales, Hymenochaetales, Polyporales, Boletales, and Agaricales) at ∼246.7 (233.1–257.3) Mya. In Auriculariales, the estimated divergence time between Exidiaceae and Auriculariaceae was ∼136.7 (113.7–171.5) Mya. *A. heimuer* was placed basal to the rest of *Auricularia* and was estimated to have a divergence time of ∼79.1 (67.4–97.1) Mya, and the estimated divergence time between *A. cornea* and *A. subglabra* was ∼54.8 (42.3–71.4) Mya (Figure 3). Further, a total of 738 gene families were significantly expanded in the *A. cornea* genome (*p* < 0.05), while only two gene families were significantly contracted. The expanded genes of *A. cornea* were mostly related to catalytic activity, metabolic processing, and proteins with binding functions, while the contracted genes were glucose repression regulatory protein TUP1 and WD40 repeat protein. With positive selection analysis, a total of 73 and 99 candidate genes were identified in *A. cornea* and *A. heimuer*, respectively (*P* < 0.01). Compared with *A. heimuer*, more genes in *A. cornea* were related to temperature adaptability, such as cellular response to oxidative stress, fungal-type cell wall organization, signal transduction, oxidation-reduction process, DNA repair/transcription protein, phosphatase activator activity, HSP70-domain-containing protein. These genes might play a crucial role in the regulation of *A. cornea* in adapting more higher temperature than *A. heimuer*.

### Implications for Biomass Decomposition by CAZyme Families

To evaluate the composition of CAZymes used in biomass degradation by AC1 and ASM, we identified the predicted protein sequences of the two genomes to the CAZyme database. AC1 possessed 460 CAZyme genes higher than ASM (316), which both contained GH, AA, PL, GT, CE, and CBM families (Supplementary Table S4 and Supplementary Figure S1). Among these, GHs and AAs were most abundant in the AC1 and ASM genomes and are primarily involved in the degradation of cellulose, hemicellulose, lignin, chitin, pectin, and starch in agro-industrial wastes. A total of 240 and 83 genes encoded GHs and AAs in the AC1 genome, respectively, while 169 and 42 were present in that of ASM, respectively. Twenty-seven expanded GH gene subgroups were identified in the AC1 genome compared to that for ASM, which included cellulose-degrading GHs (GH5, GH6, GH7, and GH12), hemicellulose-degrading GHs (GH10 and GH43), pectin-degrading GHs (GH28 and 105), chitin-degrading GHs (GH18) and starch-degrading GHs (GH31 and GH133). All AA subgroups were belonged to expanded gene families in AC1 compared to ASM. In particular, more than twice the number of AA genes encoding AA3, AA6, and AA9 were present in the AC1 genome compared to that of ASM. The numbers and types of gene encoding GTs, PLs, and CEs were similar in both AC1 and ASM genomes. Most of the CBMs observed in the two genomes were present in the N-terminal or C-terminal regions of the GH, AA, and CE family proteins. For example, genes encoding CEs and AAs contained an additional CBM1 region including CE5, CE1, CE15, AA9, and AA8 (AA12). In addition, genes encoding GHs exhibited more diversity of additional CBMs including CBM1 in GH7 and GH6, CBM5 in GH18, and CBM35 in GH43. Moreover, many CAZyme genes identified as belonging to the same subgroup were clustered in the AC1 genome. For example, two, five, and five genes encoding CE5, AA3, and GH16 genes were clustered on contigs 16, 19, and 18 of the AC1 genome, respectively.

### Genes Involved in Secondary Metabolite Biosynthesis

Terpenoids are one of the primary types of secondary metabolite (SM), which are pharmacologically active in *A. cornea*. To identify genes involved in terpenoid biosynthesis, we blasted the protein coding genes against the antiSMASH database. A total of 21 and four terpene gene clusters were identified in the AC1 and ASM genomes, respectively. Of these, one gene encoding lanosterol synthase associated with the biosynthesis of the triterpenoid ganoderic acid was shared between the AC1 and ASM genomes. Further, KEGG annotation of all predicted-proteins in the AC1 genome was yielded 26 genes encoding 18 enzymes involved in terpenoid backbone biosynthesis (ko00900) through the mevalonate pathway. Three genes encoding farnesyltransferase, squalene monooxygenase, and a squalene-hopene/tetraprenyl-β-curcumene cyclase involved in triterpenoid biosynthesis were also identified in the AC1 genome. In addition, we identified three genes encoding a trichodiene synthase participated in sesquiterpenoid biosynthesis.

We also identified Cytochrome P450 (CYP) genes associated with terpenoid biosynthesis and other fungal SMs in the AC1 genome. A total of 187 putative CYP sequences comprising 62 families were identified in the AC1 genome by querying the Fungal Cytochrome P450 Database. Among these, five genes in the CYP5144 family were predicted to be involved in triterpenoid biosynthesis. Furthermore, a number of genes were identified from CYP families that may have potential roles in fungal secondary metabolism including CYP51 (sterol 14α-demethylase) and CYP53 (benzoate 4-hydroxylase). Overall, the CYP505 family was the most abundant within the AC1 genome, comprising 17 genes that are associated with the degradation of plant cuticles in plant-associated fungi.

### Genomic Variation Among *A. cornea* and *A. heimuer* Populations

To investigate genomic variation among and within *A. cornea* and *A. heimuer* populations, we performed whole genome resequencing of 24 Wood Ear strains including 12 *A. cornea* and 12 *A. heimuer* strains. A total of 59.19 Gbp high-quality clean data were generated on the Illumina HiSeq platform, with average depth for each strain of 23×. We firstly performed the estimation of genomic variation among *A. cornea* and *A. heimuer* populations. Using the AC1 genome as a reference, after quality filtering, the overage rates of all 12 *A. cornea* strains were ranged from 79–99%, while that for the 12 *A. heimuer* strains were only 23–27% (Supplementary Figure S2A and Supplementary Table S5). Using the ASM genome as a reference, the coverage rates of all 12 *A. heimuer* strains were 87–94%, while that for the 12 *A. cornea* strains were only 19–21% (Supplementary Figure S2B and Supplementary Table S6). Thus, these results indicated the significant genomic differentiation among *A. cornea* and *A. heimuer* populations. Therefore, we further separately analyzed the genomic variations for the *A. cornea* and *A. heimuer* populations using their corresponding genomes as references.

A total of 2,403,095 high-quality SNPs were observed in the 12 *A. cornea* strains aligning the reads against the AC1 reference genome (Supplementary Figure S2C). Among these, 369,663 (15%) non-synonymous SNPs were identified within 15,409 genes. In total, 843,805 (35%) synonymous SNPs were also located within exons, yielding a non-synonymous/synonymous ratio of 0.44. In addition, 137 (0.04%) large (>100 bp) and 342,009 (99.8%) small (<50 bp) InDel polymorphisms were identified within the 12 *A. cornea* strains. Overall, 89% and 11% of the InDels were present in coding regions and non-coding regions, respectively.

A total of 1,232,179 high-quality SNPs were observed in the 12 *A. heimuer* strains aligning the reads against the ASM reference genome (Supplementary Figure S2D). Among these, 185,037 (15%) non-synonymous SNPs were identified in 12,861 genes. A total of 306,099 (25%) synonymous SNPs were located within exons, yielding a non-synonymous/synonymous ratio of 0.60. A total of 163 (0.09%) large (>100 bp) and 174,557 (99.8%) small (<50 bp) InDel polymorphisms were observed within the 12 *A. heimuer* strains. Overall, 61% and 39% of the InDels were present in coding and non-coding regions, respectively.

### Demographic History of *A. cornea* and *A. heimuer* Populations

**FIGURE 4:**
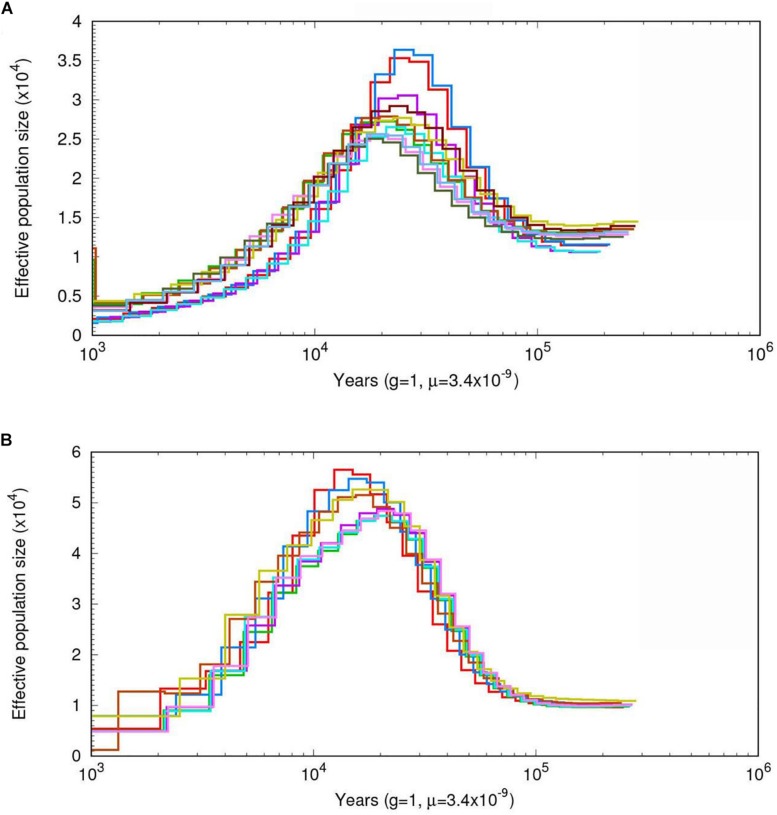
The estimated demographic histories of the two *Auricularia* populations. Diagrams show effective population sizes over time for **(A)** *A. cornea*, and **(B)** *A. heimuer*. Number at each noder epresents the time of divergence in thousands of years.

To infer the demographic histories of the ancestors of *A. cornea* and *A. heimuer*, we estimated their effective population sizes using a PSMC model. The effective population sizes of *A. cornea* and *A. heimuer* were fluctuated over time with climatic changes. The population of the ancestor of these two species were gradually expanded ∼100 kiloyears ago (Kya), with the largest effective population size of *A. cornea* and *A. heimuer* occurring ∼30–18 Kya (Figure 4A) and ∼22–13 Kya (Figure 4B), respectively. The effective population sizes of the two species both declined following these periods.

### The Genetic Differentiation in the Asia and Africa Populations of *A. cornea*

To investigate the genetic differentiation in the Asia and Africa populations of *A. cornea*, we conducted a genome-wide scan for selective sweeps for these two population and identified 249 genes. GO and KEGG annotation showed that these genes were related to temperature adaptability, such as heat shock protein 90 and 98, DNA repair protein, transcription factor SFP1, serine/threonine-protein kinase, transferase activity, and catalytic activity, indicating these genes might contribute to *A. cornea* survive in tropical and temperate regions.

### Development of Novel SSR and InDel Markers for *A. cornea*

**FIGURE 5:**
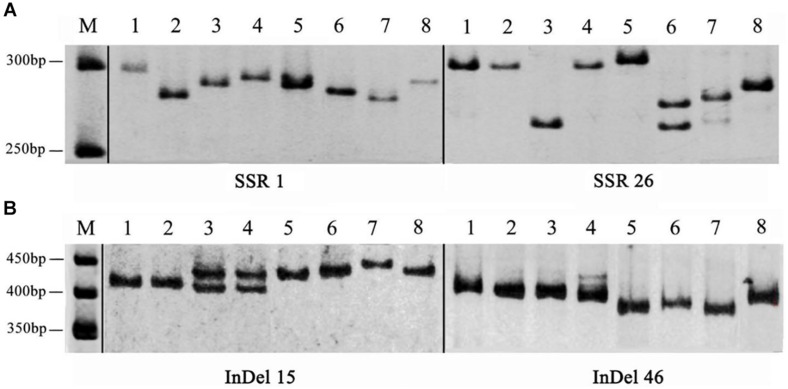
The amplification products of SSR and InDel primers developed from the *A. cornea* genome. **(A)** Polymorphic bands between eight *A. cornea* strains using SSR1 and SSR26. **(B)** Polymorphic bands between eight *A. cornea* strains using InDel15 and InDel46.

To develop novel SSR and InDel markers for *A. cornea*, we successfully designed 5,154 SSR primer pairs for *A. cornea* using the Primer 3 software. Among them, 50 primer pairs were selected to evaluate their polymorphism in *A. cornea* stains, including DNR, TNR, tetranucleotide repeats (TTNR), pentanucleotide repeats (PNR) and hexanucleotide repeats (HINR) types of SSR loci with a number of ten for each type (Supplementary Table S7). The results showed that 42 primer pairs yielded clear amplifications, and 29 primers (AcSSR1, 3–6, 8–11, 15, 17, 18, 21–27, 30, 31, 35–37, 42–44, 48, and 49) revealed polymorphic variation within the 8 *A. cornea* strains. Therefore, the overall amplification rate was 84.00% and the polymorphism rate was 58.00% (Figure 5A). We also designed 50 InDel primer pairs for *A. cornea*, which were located in exonic (i.e., frameshift insertion, non-frameshift, deletion, or stopgain) and splicing regions (Supplementary Table S8). Among them, 47 (94.00%) InDels (excluding AcInDel30, 35, and 40) successfully amplified the predicted products from all ten strains. Moreover, 35 InDel loci (70.00%, AcInDel1–10, 12–17, 21, 23, 25, 27, 29, 31–33, 36, 37, 39, 41, and 44–50) revealed polymorphisms via alleles of different sizes (Figure 5B). These newly developed primers can be further used for QTL mapping of important agronomic traits for *A. cornea*.

## Discussion

Wood Ear fungi have abundant nutritional and medicinal value and are important for the degradation of cellulose and lignin. However, the speciation, evolution, and genetic differentiation of Wood Ear are largely unknown. Only two genomes for *Auricularia* species have been previously reported including *A. subglabra* and *A. heimuer* (Floudas et al., 2012; Yuan et al., 2019). The sequenced genome of *A. subglabra* was exhibited low continuity (e.g., a scaffold N50 < 0.05 Mbp) and highly fragmented (scaffold number >1,500) (Floudas et al., 2012). *A. heimuer* was exhibited much higher sequencing quality with 103 contigs and the contig N50 of 1.35 Mbp (Yuan et al., 2019). Here, we report a high-quality genome sequence for *A. cornea* comprising 24 contigs with the contig N50 of 4.35 Mbp. The *A. cornea* genome provides an important resource for future comparative genomic and evolutionary phylogenomic studies of *Auricularia*.

Variation in genome size is one of the most striking features of eukaryotic organisms (Kelkar and Ochman, 2011). The genome sequencing has yielded new insights into the evolutionary factors underlying genome size differences. The genomes of the genus *Auricularia* exhibited remarkable size variation. The genomes of *A. cornea* (73.48 Mbp) and *A. subglabra* (74.92 Mbp) were exhibited similar sizes that were far larger than *A. heimuer* (49.76 Mbp). In addition, the genome of *A. cornea* is considerably larger than many macrofungi including *Agaricus bisporus* (30.39–31.00 Mbp) (Morin et al., 2012; Sonnenberg et al., 2016), *G. lucidum* (43.30 Mbp) (Chen et al., 2012) and *S. commune* (38.50 Mbp) (Ohm et al., 2010). To more accurately compare the genomes of *A. cornea* and *A. heimuer*, we re-annotated the *A. heimuer* ASM genome using the same platform and methods. We found the primary factors correlated to genome size differences of *A. cornea* and *A. heimuer* were included gene numbers, repetitive elements, and gene lengths. Repeat sequences in the *A. cornea* AC1 genome were comprised 15.97 Mbp, which is nearly two times higher than in the *A. heimuer* genome (8.75 Mbp) and many other fungi (Shim et al., 2016; Sipos et al., 2017). Specifically, the LTR-RT superfamilies Gypsy and Copia were substantially more diverse and abundant in the genome of *A. cornea* (8,910 vs. 5,015; 2,181 vs. 1,604), suggesting that repeat sequences, and especially transposons, contributed the most to genome size expansion. Previous investigations of plants also indicated that differences in genome sizes between species primarily depends on the amplification of repetitive sequences such as transposons (Xia et al., 2017). In particular, the expansion of LTRs is a prominent factor leading to increased genome sizes (Guan et al., 2016). In addition, the number of genes (17,591) and total gene length (44.70 Mbp) of the *A. cornea* genome were higher than in the genome of *A. heimuer* (16,402 and 35.68 Mb), thereby contributing to the observed difference in genome sizes.

Phylogenetic analysis indicated that the ancestor of *A. heimuer* diverged from other *Auricularia* was ∼79.1 (67.4–97.1) Mya during the Cretaceous Period and *A. cornea* diverged from *A. subglabra* during the Paleogene (Gradstein and Ogg, 2012). Consequently, the large-scale expansion of angiosperms coinciding with climate changes over this time also corresponds to the divergence of *Auricularia* species (Sun et al., 2011; Wang et al., 2013; Taylor, 1990). Consistent with this supposition, climate change correlated with changes in the effective population size of the ancestor of *A. cornea* and *A. heimuer* via PSMC analysis. The ancestral populations of these two species expanded ∼100 Kya during Marine Isotope Stage 5 (MIS5, 80–130 Kya), which was the last major interglacial period (Lisiecki and Raymo, 2005; Jouzel et al., 2007). During this time period, global temperatures were elevated, which could have contributed to a population expansion for these two species. After MIS5, the effective population size of *A. cornea* gradually expanded during MIS4–3 and peaked ∼30 Kya at the beginning of MIS2. The global climate gradually changed to cold and arid full-glacial conditions following MIS2, which may have limited the expansion of the two species (Lorenzen et al., 2011). However, ancestral *A. heimuer* population sizes peaked ∼22 Kya during the Last Glacial Maximum (LGM, ∼26.5–19 Kya) (Clark et al., 2009), which was later than that of *A. cornea*. This difference could be related to the lower temperature tolerance of the former species, consistent with the facts that the mycelia of *A. cornea* would grow slowly below 15°C and stop grow and die below 4–5°C, while *A. heimuer* would grow slowly below 5°C. The population sizes of the two species gradually decreased following this period, which was most likely associated with the contraction of plant species populations and reduced suitable habitat during this time (Lorenzen et al., 2011; Chen et al., 2016).

*A. cornea* and *A. heimuer* were normally cultivated using sawdust and other kinds of agro-industrial wastes. CAZymes are enzymes involved in the decomposition of lignin, cellulose, and hemicellulose (Floudas et al., 2012; Miyauchi et al., 2016). We identified higher numbers of CAZymes in the *A. cornea* AC1 genome (460) than the *A. heimuer* ASM genome (316), highlighting the potential role of the former in the processing of more diverse agro-industrial wastes. In addition, we found our newly predicted CAZymes were significantly differed from the previously reported in the ASM genome (Yuan et al., 2019). In particular, AAs (83), CEs (16), GTs (41), PLs (13), and CBMs (56) were less abundant than in the original investigation (62, 21, 29, 14, 103), but GHs (169) were significantly more abundant than previously reported (106). Therefore, different annotation methods and parameter thresholds will significantly influence results for annotation analyses. Consequently, the same annotation methodologies are necessary when performing comparative genomic analysis between species to avoid systematic biases.

As jelly fungi, *A. cornea* and *A. heimuer* prossess the similar external morphologies without differentiated stipe and cap (Supplementary Figure S2E). However, the two species exhibit strikingly different genomic properties. For example, the homologous syntenic blocks and coverage rates between AC1 and ASM were remarkably lower than those observed for other species within the same genus (Wong et al., 2015; Zhou et al., 2016; Zhang et al., 2018). Further, the across-taxa transferability rates of SSR and InDel markers were also much lower than the rate observed in *Pleurotus* species (Dai et al., 2017; Fu et al., 2017). Thus, our comparative genomic study provides a new perspective on the evolution and genetic differentiation of *Auricularia*.

## Conclusion

In this study, we present the *de novo* sequenced genome AC1 for *A. cornea*, and then performed the comparative genome analysis: (1) we demonstrate the abundances and lengths of repetitive element sequences and protein-coding genes were mainly related to the remarkable variation in genome size of the genus *Auricularia*; (2) the estimated divergence time between *A. cornea* and *A. subglabra* was ∼54.8 (42.3–71.4) Mya, which divergence with *A. heimuer* at ∼79.1 (67.4–97.1) Mya during the Cretaceous Period; (3) reveal the role of past global climate change in the long-term population dynamics of *A. cornea* and *A. heimuer*; (4) we identified a higher number of CAZyme encoding genes in the *A. cornea* AC1 genome compared to that of *A. heimuer* ASM genome, which may contribute to a potential role in the processing of more diverse agro-industrial wastes; (5) we developed novel polymorphic SSR and InDel markers for future germplasm evaluation and QTL mapping for *A. cornea*. Overall, our genomic data could provide new perspectives on the evolution and genetic differentiation of Wood Ear fungi and facilitates future breeding.

## Data Availability Statement

The datasets generated for this study can be found in GenBank https://www.ncbi.nlm.nih.gov/nuccore/RJDY00000000.

## Author Contributions

YF and YL contributed to the conceptualization of the study and the funding. YD and YF contributed to the writing and data analysis. CY and LS contributed to the genome annotation. XL and YW contributed to the sample collection and the genome extraction. BS and XZ contributed to the SSRs and InDels primers design and the screening. ZZ contributed to the reviewing and editing of the manuscript. All authors have read and approved the manuscript.

## Conflict of Interest

CY was employed by company BGI-Shenzhen. The remaining authors declare that the research was conducted in the absence of any commercial or financial relationships that could be construed as a potential conflict of interest.

## Footnotes

1 https://github.com/ruanjue/smartdenovo

2 http://www.repeatmasker.org/

3 http://www.repeatmasker.org/RepeatModeler.html

4 https://webblast.ipk-gatersleben.de/misa/

5 http://www.kegg.jp/

6 https://fungismash.secondarymetabolites.org/#!/start

7 https://www.ncbi.nlm.nih.gov/genome/?term=Tremella+fuciformis

8 http://abacus.gene.ucl.ac.uk/software/paml.html

9 https://github.com/tanghaibao/jcvi/wiki/MCscan-(Python-version)

10 http://samtools.sourceforge.net/

11 http://www.premierbiosoft.com

## Supplementary Material

FIGURE S1 Numbers of genes encoding CAZymes within the *A. cornea* and *A. heimuer* genomes.

FIGURE S2 Genotypic and phenotypic variation among *A. cornea* and *A. heimuer* populations. **(A)** Coverage rates of *A. cornea* and *A. heimuer* populations using *A. cornea* AC1 as referencing genome. **(B)** Coverage rates of *A. cornea* and *A. heimuer* populations using *A. heimuer* ASM as referencing genome. **(C)** SNPs in the *A. cornea* populations using *A. cornea* AC1 as referencing genome. **(D)** SNPs in the *A. heimuer* populations using *A. cornea* AC1 as referencing genome. **(E)** Images of the fruiting bodies of *A. cornea* and *A. heimuer*.

TABLE S1 Information of *Auricularia cornea* and *A. heimuer* strains used for whole genome resequencing.

TABLE S2 Statistic of annotated genes in *A. cornea* and *A. heimuer* genomes.

TABLE S3 Comparison of SSR loci in *A. cornea* and *A. heimuer* genomes.

TABLE S4 Distribution of the CAZymes gene families in *A. cornea* and *A. heimuer*.

TABLE S5 Summary of whole genome resequencing data of 12 *A. cornea* strains using the reference genome AC1.

TABLE S6 Summary of whole genome resequencing data of 12 *A. heimuer* strains using the reference genome ASM.

TABLE S7 Characteristics of 50 novel SSR markers developed from *A. cornea* in this study.

TABLE S8 Characteristics of 50 novel InDel markers developed from *A. cornea* in this study.
