# Supplementary figures and images for: Genomic Analyses Provide Insights Into the Evolutionary History and Genetic Diversity of Auricularia Species
Source: Front Microbiol. 2019 Oct 1;10:2255. doi: 10.3389/fmicb.2019.02255 (PMC6786273; doi:10.3389/fmicb.2019.02255)

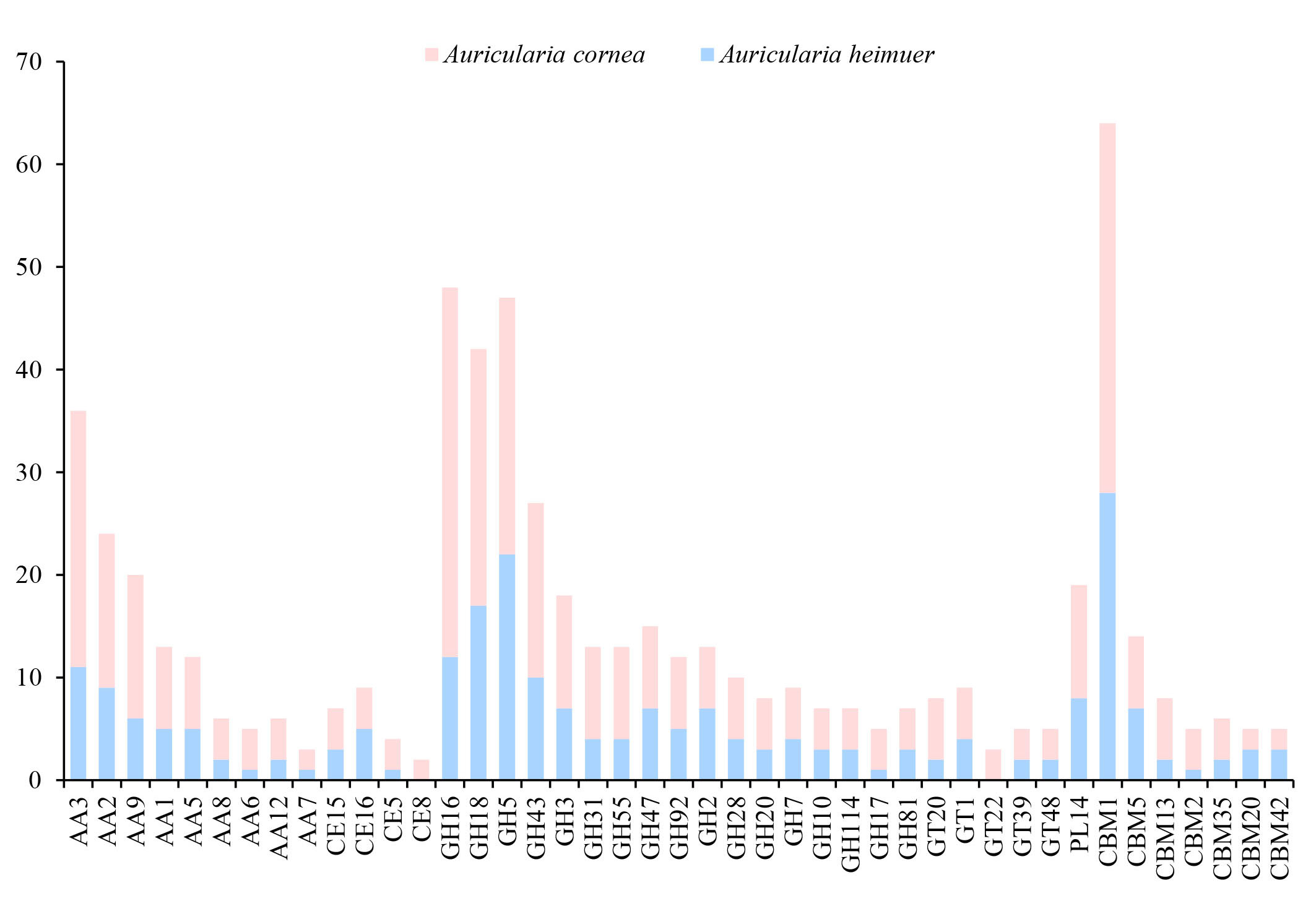

Supplement: FIGURE S1 — Numbers of genes encoding CAZymes within the A. cornea and A. heimuer genomes. [file Image_1.JPEG]

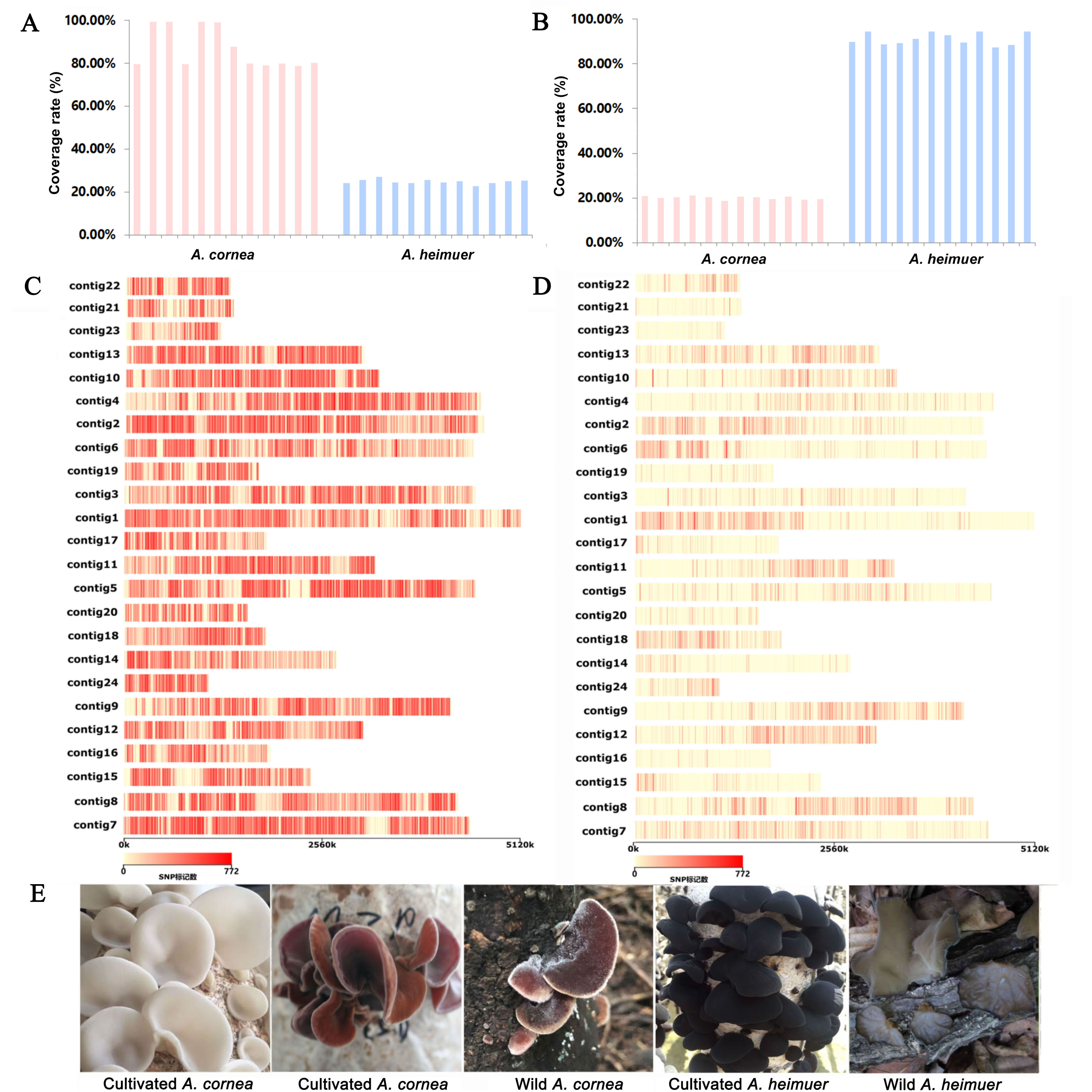

Supplement: FIGURE S2 — Genotypic and phenotypic variation among A. cornea and A. heimuer populations. (A) Coverage rates of A. cornea and A. heimuer populations using A. cornea AC1 as referencing genome. (B) Coverage rates of A. cornea and A. heimuer populations using A. heimuer ASM as referencing genome. (C) SNPs in the A. cornea populations using A. cornea AC1 as referencing genome. (D) SNPs in the A. heimuer populations using A. cornea AC1 as referencing genome. (E) Images of the fruiting bodies of A. cornea and A. heimuer. [file Image_2.JPEG]
